# Imagine-Self Perspective-Taking and Rational Self-Interested Behavior in a Simple Experimental Normal-Form Game

**DOI:** 10.3389/fpsyg.2017.01557

**Published:** 2017-09-11

**Authors:** Adam Karbowski, Michał Ramsza

**Affiliations:** ^1^Department of Business Economics, Warsaw School of Economics Warsaw, Poland; ^2^Department of Mathematics and Mathematical Economics, Warsaw School of Economics Warsaw, Poland

**Keywords:** imagine-self perspective-taking, rational behavior, self-interested behavior, empathy, normal-form games

## Abstract

The purpose of this study is to explore the link between imagine-self perspective-taking and rational self-interested behavior in experimental normal-form games. Drawing on the concept of sympathy developed by Adam Smith and further literature on perspective-taking in games, we hypothesize that introduction of imagine-self perspective-taking by decision-makers promotes rational self-interested behavior in a simple experimental normal-form game. In our study, we examined behavior of 404 undergraduate students in the two-person game, in which the participant can suffer a monetary loss only if she plays her Nash equilibrium strategy and the opponent plays her dominated strategy. Results suggest that the threat of suffering monetary losses effectively discourages the participants from choosing Nash equilibrium strategy. In general, players may take into account that opponents choose dominated strategies due to specific not self-interested motivations or errors. However, adopting imagine-self perspective by the participants leads to more Nash equilibrium choices, perhaps by alleviating participants’ attributions of susceptibility to errors or non-self-interested motivation to the opponents.

## Introduction

Weizsäcker (2003) coined the hypothesis that decision-makers’ tendency to ignore their opponents’ incentives in experimental normal-form games is an artifact of the experimental environments in the laboratories, and in particular of the use of abstract payoff matrix presentations in experimental procedures. Weizsäcker (2003) further suggests that adding a context to the experiments (and probably developing a more realistic sense of strategic choice) would help the subjects perceive their opponents’ decision problems more vividly and clearer. From the viewpoint of game-theoretic models of quantal response (see, e.g., McKelvey and Palfrey, 1995), the subjects’ tendency to ignore their opponents’ incentives can be referred to as an anomaly (Weizsäcker, 2003).

Our article takes the outlined behavioral “anomaly” of ignoring rationality of others in experimental normal-form games as a point of departure. Complementing Weizsäcker’s suggestion that developing a more realistic sense of strategic choice would help the players perceive their opponents’ decision problems clearer, and drawing on Adam Smith’s concept of sympathy (for an elaboration, please see paragraphs below in this section), we set out to investigate whether the subjects’ tendency to ignore their opponents’ incentives can be alleviated due to introduction of imagine-self perspective-taking (Stotland, 1969) by decision-makers. To this end, we examined subjects’ behavior in a simple experimental normal-form game (for details, see Materials and Methods), in which one of the two players (row player) can suffer a monetary loss if and only if she plays her Nash equilibrium (pure) strategy and the other player (column player) plays her strictly dominated (pure) strategy.

In his seminal study, Stotland (1969) distinguished two different forms of empathic perspective-taking, i.e., (1) imagine-self and (2) imagine-other. Imagine-self perspective means imagining what one’s own thoughts and emotions would be if one were in the situation of the other person (Batson, 2014). Imagine-other perspective translates in turn to imagining the thoughts and emotions of the other person (Batson, 2014). Stotland (1969) found that both above-mentioned forms of imagining lead to increased emotional arousal in comparison to adopting emotionally cool, objective perspective (Batson, 2014). Batson et al. (1997) report that imagine-self perspective-taking produces both self-oriented and other-oriented emotions in decision-makers. Imagine-other perspective-taking seems in turn to produce solely other-oriented concern (Batson, 2014).

For clarity, it is important to distinguish here between perspective-taking and empathy itself. The psychological literature reports evident differences between the above-mentioned terms (cf., e.g., Davis, 1983; Oswald, 1996; Galinsky et al., 2008; Hilliard et al., 2016). Empathy always involves emotional response which allows to affectively connect with another person, while perspective-taking is a cognitive capacity which allows to consider the world from other points of view (Galinsky et al., 2008). As Montgomery (1994) notices, in cognition different perspectives are elicited by identification with certain persons (with oneself or some other person) or with certain interests (e.g., economic interests). Importantly, perspective-taking can (but not necessarily has to) ultimately lead to formation of emotions and empathy itself. Perspective-taking that does not lead to affect formation can act as an effective cognitive device which increases individual’s abilities to perform in strategic interactions, e.g., through detecting of hidden agreements, more frequent engaging in recursive reasoning that is predictive, or reducing ambiguity (for details, please see Hanna et al., 2003; Galinsky et al., 2008; Zhang et al., 2012; Cane et al., 2017).

Apart from the cognitive links between perspective-taking and decision making, the psychological literature also reveals interesting relationships between perspective-taking and motivated behavior. An interesting example is here the study by Epley et al. (2006), which establishes the link between perspective-taking and observed selfishness. Epley et al. (2006) show that leading people to consider other persons’ thoughts may increase selfish behavior (in the cited study, perspective-taking increases taking of resources in social dilemmas). It is also worth noticing here that selfish motivations prevail over perspective-taking, as Evans and Krueger (2011) report in their study based on the experimental sequential trust game. These authors show that trust increases when costs decrease and benefits increase. Then, increasing the trustor’s benefit also means increasing the trustee’s temptation to defect. Hence, selfish motivation seems to prevail over perspective-taking.

As Grohn et al. (2014) notice, the concept of perspective-taking and empathy, despite its importance in Hume (1739) and Smith (1759) philosophical enunciations, has never gained a decent foothold in economic theory. Adam Smith’s concept of man underlines two intertwined motives in human behavior, i.e., self-interest and sympathy (1759). Self-interest is certainly, in Adam Smith’s view, a very powerful motive, but it is by no means the only motive. The inclusion of sympathy in Smith’s analysis does not weaken but rather strengthens “invisible hand” argument (Coase, 1976; Smith, 1776). In Smith’s (1759, 1776) concept, sympathy may then promote rational and self-interested behavior.

According to Smith (1759) sympathy allows us to form ideas of how others think and feel by considering how we would think and feel in like circumstances (Coase, 1976). By an act of imagination, we put ourselves in their place, and, in effect, in our own minds become those other persons (Coase, 1976). As we can see, Smithian sympathy can be interpreted as an imagine-self form of empathy.

In the following paper, we explore the link between imagine-self perspective-taking by decision-makers and their rational self-interested behavior, which is reflected by selecting Nash equilibrium strategies. It turns out that imagine-self perspective-taking by participants allows to obtain significantly more outcomes which would be attained in a group consisting only of members who behave in a sufficiently rational and self-interested manner. This result is in line with the research on reactive egoism (Epley et al., 2006), since also in our study, perspective-taking seems to lead to increase in selfish behavior.

The remainder of the paper proceeds as follows. We describe participants, materials, and experimental procedure in the next section. The results presented and analyzed in section “Results” are then briefly discussed in the light of relevant psychological and economic theories in the final section.

## Materials and Methods

### Overall Design of Experiment

To test expectations elaborated upon in the previous section, an experiment was designed and conducted. The experimental procedure comprised a single two-player normal-form game. The game was presented to four experimental groups:

• group 1 – “row players without imagine-self instructions,”
• group 2 – “column players without imagine-self instructions,”
• group 3 – “row players with imagine-self instructions,”
• group 4 – “column players with imagine-self instructions,”

numbering 105, 104, 96, and 99 subjects, respectively.

Each group, prior to solving the game, was given different instructions. Groups 1 and 2 received instructions without imagine-self-related task and groups 3 and 4 with imagine-self exercise. The content of instructions, that is, “with” or “without” imagine-self exercise, constitutes an independent variable in the experiment. The full set of instructions is presented further in this section, see **Table 2**.

As with imagine-self perspective-taking people try to imagine themselves in other people’s shoes, instructions given to groups 3 and 4 are intended to induce imagine-self perspectives in subjects belonging to those groups (imagine-self manipulation). The dependent variable is the choice of strategies made by the participants in the proposed game.

The research (before the study began) was reviewed and approved by the review board of the Warsaw School of Economics. Written informed consents were obtained from the participants.

### Participants

Participants of the experiment were 404 undergraduate students of the Warsaw School of Economics (SGH). Precisely, first year and second year students participated in the experiment.

### Materials

During the experiment the following two-player normal-form game was used (**Table 1**).

**Table 1:** The normal-form game used in the experiment.

	L	R
T	600; 600	-300; 500
B	500; 600	300; 500

*Source: Own material*.

Note that the game in **Table 1** is solvable through the process of iterative elimination of the strictly dominated strategies. It is easy to see that strategy R of a column player is strictly dominated by strategy L, that is, regardless of a row player’s choice, strategy L yields higher payoff. Also, the payoff of a column player does not depend on a row player’s choice. Once strategy R is eliminated, strategy B of a row player becomes strictly dominated. Consequently, there is a single strict pure strategy Nash equilibrium (T, L).

Each group was given a different set of instructions. Exact instructions are given in **Table 2**.

**Table 2:** Experimental instructions.

Group number	Instructions given
1	You are a row player (you choose between T and B). Indicate your choice by underlining one of the following strategies: T or B.
2	You are a column player (you choose between L and R). Indicate your choice by underlining one of the following strategies: L or R.
3	You are a row player (you choose between T and B). Before you make your choice, what would be your choice if you were a column player: L or R (indicate your choice by underlining). Now make choice for yourself: T or B (indicate by underlining).
4	You are a column player (you choose between L and R). Before you make your choice, what would be your choice if you were a row player: T or B (indicate your choice by underlining). Now make choice for yourself: L or R (indicate by underlining).

*Source: Own material*.

Instructions for groups 1 and 2 did not involve any suggestions related to imagine-self perspective-taking. They were simple instructions assigning the role, either of row player for a group 1 or column player for a group 2, to a subject and asking about a choice in the assigned role. Instructions given to groups 3 and 4 contained additional tasks related to imagine-self perspective-taking. Precisely, for subject assigned a role of row player the task was to imagine what would be her choice if she were a column player, and the other way around for a subject assigned a role of column player. The goal of such manipulation was to induce imagine-self perspective-taking of participants.

### Experimental Procedure

Participants did not have any prior knowledge about games. Therefore, a short (about 10 min) tutorial was given at the beginning of the procedure. It was then checked that the participants understood a concept of a player, strategy, and payoff. Specifically, it was checked that the connection between the choice of a strategy and a payoff is clear.

The subjects were told to think of numbers in **Table 1** as if they were monetary amounts in euros (these are significant monetary values for the SGH undergraduate students; 500 euros cover average monthly living expenses of the SGH undergraduate students) that the participants can gain (positive payoffs) or lose (negative payoffs) depending on the players’ decisions taken in the game.

After the tutorial, participants were given randomly chosen versions of printed instructions (one of four versions, see **Table 2**) with the normal-form game. The content of instruction was private knowledge, subjects did not know instructions given to others. The solving of the game by participants was not time-limited, and on average it took a participant about 3 min to indicate her/his choice. The printed instructions were next collected and the results were aggregated in a spreadsheet application.

In this study, we decided to formulate the following research hypotheses. They form two identical sets of three hypotheses, one for role of row player, hypotheses 1–3, and one for role of column player, hypotheses 4–6. The hypotheses are followed by brief justification.

Hypothesis 1 In the first experimental group (row players without imagine-self instructions) a proportion of subjects choosing strategy B is higher than a proportion of subjects choosing strategy T.
Hypothesis 2 In the third experimental group (row players with imagine-self instructions) a proportion of subjects choosing strategy T is higher than a proportion of subjects choosing strategy B.
Hypothesis 3 A proportion of subjects choosing strategy T in the third experimental group (row players with imagine-self instructions) is higher than a proportion of subjects choosing strategy T in the first experimental group (row players without imagine-self instructions).
Hypothesis 4 In the second experimental group (column players without imagine-self instructions) a proportion of subjects choosing strategy L is higher than a proportion of subjects choosing strategy R.
Hypothesis 5 In the fourth experimental group (column players with imagine-self instructions) a proportion of subjects choosing strategy L is higher than a proportion of subjects choosing strategy R.
Hypothesis 6 A proportion of subjects choosing strategy L in the fourth experimental group (column players with imagine-self instructions) is equal to a proportion of subjects choosing strategy L in the second experimental group (column players without imagine-sel instructions).

Observe that in our simple experimental normal-form game the row player can suffer a monetary loss if and only if she plays her Nash equilibrium (pure) strategy T and the other player plays her dominated (pure) strategy R. We think that the threat of suffering monetary losses can effectively discourage the row players from choosing strategy T in the first experimental group – “row players without imagine-self instructions,” thus hypothesis 1 is directional and the alternate testing hypothesis is also directional.

The row player can suffer monetary losses while playing Nash strategy T if and only if column player does not conform to the strict Nash equilibrium of the game. From the point of view of a row player, this may have two sources. Firstly, a row player can completely neglect considering an opponent, resulting in making decisions under ambiguity. Secondly, a row player can rationally take the possibility of playing strategy R by column players into account. The column players can play strategy R either because of their not full rationality [following Hendrikse (2003), full rationality means that the ratio of decision-maker’s cognitive capacities to problem complexity always equals 1; consequently, a decision-maker is able to immediately solve any problem and makes no mistakes] or their specific not self-interested motivation. However, in the third experimental group (row players with imagine-self instructions), drawing on Adam Smith’s concept of man and further literature on perspective-taking (see section “Introduction”), we hypothesize that, first, solving the game from the point of view of a column player clearly forces the subject in a role of row player to actively consider potential choices of an opponent. Second, adopting imagine-self perspective may alleviate attributing (by row players) a susceptibility to errors or some non-self-interested motivations to the column players and, in effect, promote rational self-interested behavior. As a result, in the third experimental group (row players with imagine-self instructions), we hypothesize that the proportion of subjects playing T is higher than the proportion playing B, hence hypothesis 2 is directional. Also, we conjecture that it is the imagine-self perspective-taking-related instruction that makes the difference, thus we formally test hypothesis 3.

Since in our study column players are not faced with the threat of suffering monetary losses and their payoffs do not depend on row player’s strategies, adopting imagine-self perspective by the column decision-makers should not change much in their choices. L should be a desirable strategy no matter how the row player is perceived and if at all. This reasoning stands behind hypotheses 4–6.

There is one additional reason to include experimental group 2. None of the subjects received any monetary reward for taking part in the experiment. Then, it is a valid question to ask if participants attached any value to the payoffs given in the problem matrix. The task of the second experimental group is to control for that. If majority of subjects in the second experimental group select the beneficial strategy L, then it suggests that clearly attention is paid to the payoffs in the problem matrix.

## Results

**FIGURE 1:**
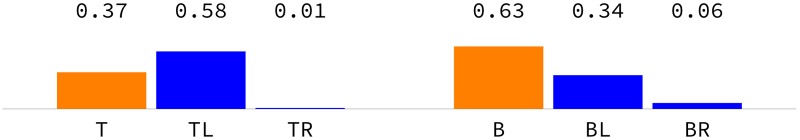
Relative frequencies of choices made in experimental groups 1 (orange) and 3 (blue).

**FIGURE 2:**
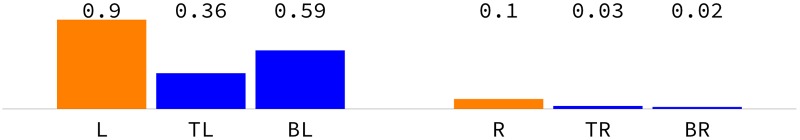
Relative frequencies of choices made in experimental groups 2 (orange) and 4 (blue).

**FIGURE 3:**
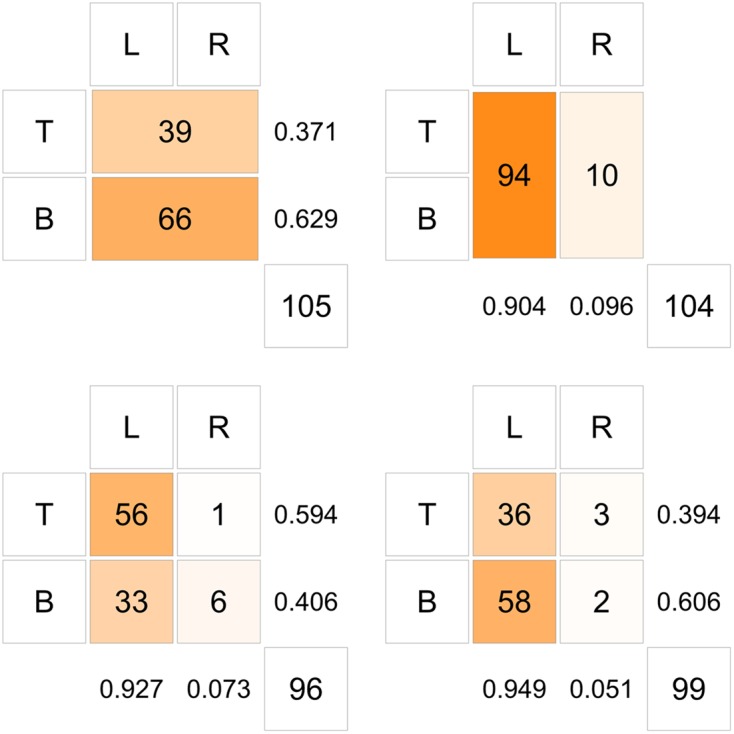
Results received aligned with the structure of the experimental game.

The results of the experiment are given in **Table 3**. For better display, results of the experiment are also depicted as bar charts, see **Figures 1**, **2**, and as a diagram aligned with the structure of the experimental game, see **Figure 3**. In **Figure 3**, marginal distributions are also provided.

**Table 3:** Results received.

Strategy chosen	Number of participants that chose the given strategy	Relative frequency of the given choice
**Group 1**		
T	39	0.371
B	66	0.629
**Group 2**		
L	94	0.904
R	10	0.096
**Group 3**		
TL (the two-letters notation indicates participants’ choices made in the third and fourth experimental groups)	56	0.583
TR	1	0.010
BL	33	0.344
BR	6	0.063
**Group 4**		
TL	36	0.364
TR	3	0.030
BL	58	0.586
BR	2	0.020

*Source: Own material*.

Observe that for the first two (**Table 2**) experimental groups (players without imagine-self instructions), only one strategy is to be selected (one choice is to be made), but for the last two experimental groups (players with imagine-self instructions) two strategies make up an answer (two choices are to be made by participants) and so there are four possibilities.

Some of the results seem clear. In the first experimental group (row players without imagine-self instructions) only about 37% of subjects chose strategy T and about 63% went with a choice of strategy B. This result seems not even close to the unique strict pure strategy Nash equilibrium of the presented game. Almost all (over 90%) participants of the second experimental group (column players without imagine-self instructions) chose the dominant strategy L. Only 10 out of 104 subjects chose the dominated strategy R.

In the third experimental group (row players with imagine-self instructions) over 90% of subjects chose the Nash equilibrium strategy for a column player (strategy L) and at the same time about 59% of subjects chose the Nash equilibrium strategy T. This share is visibly higher than in the first experimental group (about 59% in the third group to about 37% in the first one). In the fourth experimental group (column players with imagine-self instructions) about 95% of subjects chose the Nash equilibrium strategy of a column player (in comparison to about 90% in the second group, respectively). In the fourth experimental group about 39% of subjects chose the Nash equilibrium strategy of a row player (in comparison to 37% in the first group, and almost 60% in the third experimental group).

Turning to our research hypotheses, for testing about a single proportion, an exact test of the statistical significance of deviations from a theoretically expected distribution of observations into two categories was used (binomial test), and for testing about two proportions, a permutation (randomization) test was used. The results of the tests are given in the following **Table 4**. The *p*-values reported in **Table 4** refer to null testing hypotheses (second column).

**Table 4:** Test results.

Research hypothesis	Null testing hypothesis	*p*-Value	Alternate testing hypothesis
1	Probability of choosing strategy T is 0.5	0.005409^∗∗^	Probability of choosing strategy T is smaller than 0.5
2	Probability of choosing strategy T is 0.5	0.0411^∗^	Probability of choosing strategy T is greater than 0.5
3	Probability of choosing T in the third group is equal to probability of choosing T in the first group	0.001263221^∗∗^	Probability of choosing T in the third group is higher than probability of choosing T in the first group
4	Probability of choosing L is 0.5	2.2*e*-16^∗∗∗^	Probability of choosing L is greater than 0.5
5	Probability of choosing L is 0.5	2.2*e*-16^∗∗∗^	Probability of choosing L is greater than 0.5
6	Probability of choosing L in the fourth group is equal to probability of choosing L in the second group	0.2854253	Probability of choosing L in the fourth group is not equal to probability of choosing L in the second group

*Comment: Stars next to p–values indicate significance level; Source: Own material*.

## Discussion

Looking for explanations of the observed behavior of row players in our experiment, we think of the following factors. The first explanation relates to the hypothesis coined by Weizsäcker (2003) that states that decision-makers simply ignore any incentive structure of opponent. In this case it is quite possible that row players choose to maximize the worst payoff, that is, they play the maxmin strategy resulting in selecting strategy B. As already mentioned, the row players may also choose non-Nash equilibrium strategy because of rational expectations that the column player is not fully rational or is motivated to act in a not self-interested manner. The latter can be at least partially explained by social value orientation (SVO) theory (McClintock, 1972; Griesinger and Livingston, 1973). For example, the row player may know that the column player exhibits competitive orientation, and so seeks for a maximization of her relative gain. Note that selecting strategy R by the column player may, in fact, mean sacrificing risk-free 100 euros to inflict losses on the row player. However, the choice of strategy R gives chance to maximize the difference between the payoff received by the column and row player. Certainly, the self-interested column decision-maker would instead select strategy L to maximize her individual gain.

The row player may also take the possibility of mistake made by the column player into account. Remember that in neoclassical economics full rationality occurs when the ratio of decision-maker’s cognitive capacities to problem complexity always equals 1. Consequently, a decision-maker is able to immediately solve any problem and makes no mistakes. Since this form of rationality is postulative in nature and not realistic (see, e.g., Selten, 1999), the row players may convincingly take the possibility of errors made by column players into account. The column players may do not understand the decision problem properly, make mistakes in solving the problem, or make mistakes in indicating the desired answer. Simply put, the row players may attribute some other form of rationality (other than full rationality) to column players, i.e., bounded (limited) rationality or procedural rationality (cf., Hendrikse, 2003). However, if the probability that a column player chooses strategy R is not larger than 1/7 ≈ 0.143, it is always better (in terms of expected payoff) to choose strategy T. If row players correctly predict the error rate, which in our experiment never exceeded 0.1, they should always use strategy T to maximize expected payoff. Thus, for this explanation to be correct, row player should grossly overestimate the error rate.

Regarding subjects’ attributions, it is also worth stressing that the only measure used in the current study is behavior, which in this case does not strongly constrain the psychological interpretation. That is, the observed behavior could, but need not necessarily, have derived from changes in row players’ attributions of error susceptibility to column players. Therefore, let us underline that our inference about players’ attributions is only indirect.

By referring to the perspective theory of decision making (Montgomery, 1994), we may now propose an explanation of the shift in behavior of row players between conditions 1 and 3 (“row players without imagine-self instructions” versus “row players with imagine-self instructions”). As Montgomery (1994) notes, dependent on the congruence between subject orientation and characteristics of the object, different perspectives can be adopted by decision-maker. When the object is seen as separate from the subject, an outside perspective is adopted and negative features dominate the perception. When the object is seen as affiliated with the subject, an inside perspective is adopted and positive features dominate the perception. We think that imagine-self manipulation allowed to shift the perspective taken by at least some participants from an outside to an inside perspective. Under outside perspective the threat of monetary losses (-300) dominated the subjects’ perception (so the choice was B), however, under inside perspective the highest possible gain for oneself (600) dominated (so the choice was T).

It is also worth observing that our results regarding the behavior of row players seem consistent with the research by Epley et al. (2006) who stated that, paradoxically, leading people to consider other persons’ thoughts may actually increase selfish behavior such that people take more of available resources.

Let us now comment on the results corresponding to the hypotheses (4–6) concerning the behavior of the column players. As expected, the adopting of imagine-self perspective by column decision-makers does not change much in their choices. L remains a desirable strategy no matter how the row player is perceived. It is, however, worth noting that the observed relative frequency of L choices is higher in the fourth experimental group (column players with imagine-self instructions) than in the second one (column players without imagine-self instructions): 0.949 versus 0.904, though the differences are not statistically significant. However, the direction of this shift does not violate the interpretations offered by perspective theory of decision making (Montgomery, 1994), because the positive feature (the highest possible gain to attain – 600), after imagine-self manipulation, beats the other option (500) even more.

It also worth noticing that when column players are asked to put themselves in row players’ shoes, column players think row player would go by monetary losses and select strategy B. It means that the results of the game equipped with bilateral imagine-self instructions (experimental conditions 3 and 4 taken together) would be surprising for the column players, but not for the row players who faced a threat of monetary losses. Perhaps then, in strategic interactions, imagine-self perspective-taking brings significant benefits in predictive reasoning (Zhang et al., 2012) to the decision-maker who is at the risk of losses. The latter has to be a subject of further investigation.

Additionally, when column players are asked to put themselves in row players’ shoes, column players predict that around 0.39 of row players choose strategy T, that is very close to the result in the first experimental group. At the same time, this estimate is too low for the results obtained in the third experimental group. This may suggest that in the fourth experimental group subjects answered the first question in the similar way as subjects in the first experimental group. On the other hand, predictions regarding choices of column players done by row players in the third experimental group are very close to results obtained in the second and fourth group, in fact, the prediction is almost an average of the two groups. We may thus conclude that predictions of row players regarding behavior of column players are very accurate. It may suggest that behavior of row players in the first experimental group is rather a result of ignoring opponents than considering errors or other SVO of column players, and the observed difference in behavior between the first and the third group is due to induced switch in perspective.

Finally, when we look at the results, we can conclude that in our experimental game, the pure strategy Nash equilibrium is not the best predictor of the empirical results. Note that in a game without imagine-self instructions, most outcomes are (B, L) instead of the single strict pure strategy Nash equilibrium (T, L). The pure strategy Nash equilibrium concept works better when assisted with the experimental instructions intended to induce imagine-self perspective. In a game equipped with such instructions, most outcomes are (T, L).

Interestingly, psychological research (see, e.g., Batson, 2011) indicates strong links between imagine-other perspective-taking and motivated behavior (the imagine-other-altruism hypothesis), links that are not present when decision-makers adopt imagine-self perspective (Grohn et al., 2014). But, since Nash equilibrium concept is believed to model self-interested behavior of decision-makers involved (Cohen, 1998), and the following study links imagine-self perspective-taking to Nash equilibrium behavior, the imagine-self–self-interest hypothesis could be also considered.

Our findings complement Weizsäcker’s (2003) suggestion that developing a more realistic sense of strategic choice would alleviate decision-makers’ tendency to ignore their opponents’ rational self-interested behavior in experimental normal-form games. We show that introduction of imagine-self instructions into our experimental game allows to promote rational self-interest of decision-makers. We also experimentally test Smith’s (1759, 1776) conjecture that imagine-self perspective-taking (or sympathy, in Smithian terms) may promote rational self-interested behavior. Based on our experiment, we cannot falsify Smith’s hypothesis. Lastly, our results are in line with the research on reactive egoism (Epley et al., 2006), since also in our study, perspective-taking seems to lead to increase in selfish behavior. However, our study, in contrast to research by Epley et al. (2006), is based on experimental normal-form game in which participants cannot communicate with each other. Epley’s experiments were much more interactive.

## Author Contributions

AK conception and design of the work, data collection, interpretation of the data, and drafting the work and MR conception and design of the work, analysis of the data, revising the work, and ensuring accuracy and integrity of any part of the work.

## Conflict of Interest Statement

The authors declare that the research was conducted in the absence of any commercial or financial relationships that could be construed as a potential conflict of interest.
